# Risk assessment of imported malaria in China: a machine learning perspective

**DOI:** 10.1186/s12889-024-17929-9

**Published:** 2024-03-20

**Authors:** Shuo Yang, Ruo-yang Li, Shu-ning Yan, Han-yin Yang, Zi-you Cao, Li Zhang, Jing-bo Xue, Zhi-gui Xia, Shang Xia, Bin Zheng

**Affiliations:** 1 National Institute of Parasitic Diseases, Chinese Center for Disease Control and Prevention (Chinese Center for Tropical Diseases Research); NHC Key Laboratory of Parasite and Vector Biology; WHO Collaborating Centre for Tropical Diseases; National Center for International Research on Tropical Diseases, Shanghai, 200025 China; 2 National Key Laboratory of Intelligent Tracking and Forecasting for Infectious Diseases, National Institute of Parasitic Diseases at Chinese Center for Disease Control and Prevention, Chinese Center for Tropical Diseases Research, Shanghai, 200025 China; 3 School of Global Health, Chinese Center for Tropical Diseases Research, Shanghai Jiao Tong University, School of Medicine, Shanghai, 200025 China

**Keywords:** Imported Malaria, Risk Assessment, Risk prediction, Risk mapping, Machine learning, Random Forest, China

## Abstract

**Background:**

Following China’s official designation as malaria-free country by WHO, the imported malaria has emerged as a significant determinant impacting the malaria reestablishment within China. The objective of this study is to explore the application prospects of machine learning algorithms in imported malaria risk assessment of China.

**Methods:**

The data of imported malaria cases in China from 2011 to 2019 was provided by China CDC; historical epidemic data of malaria endemic country was obtained from World Malaria Report, and the other data used in this study are open access data. All the data processing and model construction based on R, and map visualization used ArcGIS software.

**Results:**

A total of 27,088 malaria cases imported into China from 85 countries between 2011 and 2019. After data preprocessing and classification, clean dataset has 765 rows (85 * 9) and 11 cols. Six machine learning models was constructed based on the training set, and Random Forest model demonstrated the best performance in model evaluation. According to RF, the highest feature importance were the number of malaria deaths and Indigenous malaria cases. The RF model demonstrated high accuracy in forecasting risk for the year 2019, achieving commendable accuracy rate of 95.3%. This result aligns well with the observed outcomes, indicating the model’s reliability in predicting risk levels.

**Conclusions:**

Machine learning algorithms have reliable application prospects in risk assessment of imported malaria in China. This study provides a new methodological reference for the risk assessment and control strategies adjusting of imported malaria in China.

## Introduction

Malaria, tuberculosis, and HIV/AIDS are widely recognized as significant global public health challenges. Malaria exhibits a longstanding prevalence within the borders of China, encompassing a broad range of impact and severe consequences, thereby positioning it as one of the most consequential infectious diseases. For a considerable duration, this issue has presented a substantial risk to the well-being and security of the Chinese populace, exerting a notable impact on China’s socioeconomic progress [1]. China successfully achieved the milestone of zero indigenous cases of malaria in 2017, following years of dedicated endeavors aimed at eliminating the disease. In 2021, China was officially granted the World Health Organization’s (WHO) certification of being malaria-free [2]. Nevertheless, it is important to acknowledge that numerous countries across the globe continue to experience endemic malaria, leading to a substantial loss of lives. Based on the findings presented in the “World Malaria Report 2022” by the World Health Organization, it is estimated that there were approximately 247 million instances of malaria worldwide in the year 2021, distributed across 84 different countries or regions. The vast majority of malaria cases is primarily concentrated in Africa and Southeast Asia, wherein 29 nations collectively contribute to 96% of the worldwide malaria cases [3].

The economic growth experienced by China in recent years, coupled with the enhanced global trade activities, particularly following the introduction of policies such as the “Belt and Road Initiative,” has led to a notable surge in the number of Chinese citizens engaging in international travel for purposes including employment, tourism, business, and cross-cultural interactions. Nevertheless, the heightened probability of malaria importation into China from foreign sources has emerged as a formidable obstacle to the preservation of the progress achieved in malaria eradication efforts within the nation [4]. Prior to the onset of the Covid-19 pandemic, China documented an annual average of more than two thousand instances of imported malaria, originating from various countries in Africa and Southeast Asia, among other regions. After the Chinese government implemented measures to ease international travel restrictions and enforce strict border controls, there was a gradual restoration of international flights and resumption of population movements. The aforementioned circumstances have presented considerable obstacles to the implementation of preventive, control, and quarantine strategies aimed at addressing imported cases of malaria and other infectious diseases. The implementation of risk assessments on populations that are potentially at risk and countries that are considered high-risk sources can be an effective strategy in mitigating the risk of retransmission of imported malaria within China [5]. In response to the emerging obstacles encountered during the post-elimination stage of malaria control, pertinent entities in China, including the Chinese Center for Disease Control and Prevention (China CDC) and the National Health Commission, have put forth the approach of “timely detection and precise prevention” as a strategic measure. This approach places emphasis on the monitoring and observation of activities, with a particular focus on the core principles of timely identification, prompt notification, and swift intervention. The primary objective is to expeditiously detect and scientifically intervene in instances of imported malaria, thereby guaranteeing timely and focused measures to mitigate the spread of the disease [6]. The implementation of thorough risk assessments pertaining to the retransmission of imported malaria is of utmost importance in order to effectively prevent its reintroduction. Hence, it is imperative to undertake precise identification and conduct a comprehensive risk assessment of the countries from which malaria is imported. This procedure has the potential to yield significant insights for the formulation of public health policies and the distribution of resources intended to prevent and manage cases of imported malaria [7].

The utilization of machine learning models exhibits significant potential in the field of infectious disease surveillance and timely alert systems. Supervised learning is a commonly utilized algorithm within the field of machine learning, wherein models are trained using annotated data to facilitate predictions and classifications on unfamiliar data. Supervised learning commonly employs various algorithms, such as Random Forests (RF), Support Vector Machines (SVM), and artificial neural networks (ANN), among others. The aforementioned algorithms possess the capability to proficiently examine patterns and correlations within data, thereby facilitating precise forecasting and categorization of outbreaks of infectious diseases [8–11]. Machine learning has made significant contributions to various impactful studies conducted in the domain of malaria research [12–21]. Especially SVM and decision tree algorithms, which have been applied by researchers such as Michel Olaolu Arowolo to the study of malaria [22–23]. Nevertheless, it is important to acknowledge that a significant portion of these studies have predominantly concentrated on the supplementary identification of malaria instances. There has been a paucity of research conducted on the assessment and prediction of risk factors associated with malaria in various countries.

This study adopts a socio-economic and epidemiological framework to examine the risk factors linked to imported malaria cases in China. Machine learning models are developed by utilizing data from the years 2011 to 2019, encompassing various factors such as the amount of imported malaria cases in China, the prevalence of malaria in the source countries, the population size of the source countries, the economic status of these countries, and the level of trade interactions between China and the countries posing a risk. In this study, six supervised learning algorithms were utilized to investigate the potential utilization of machine learning techniques in predicting the risk level of source countries for imported malaria cases in China.

**Fig. 1:**
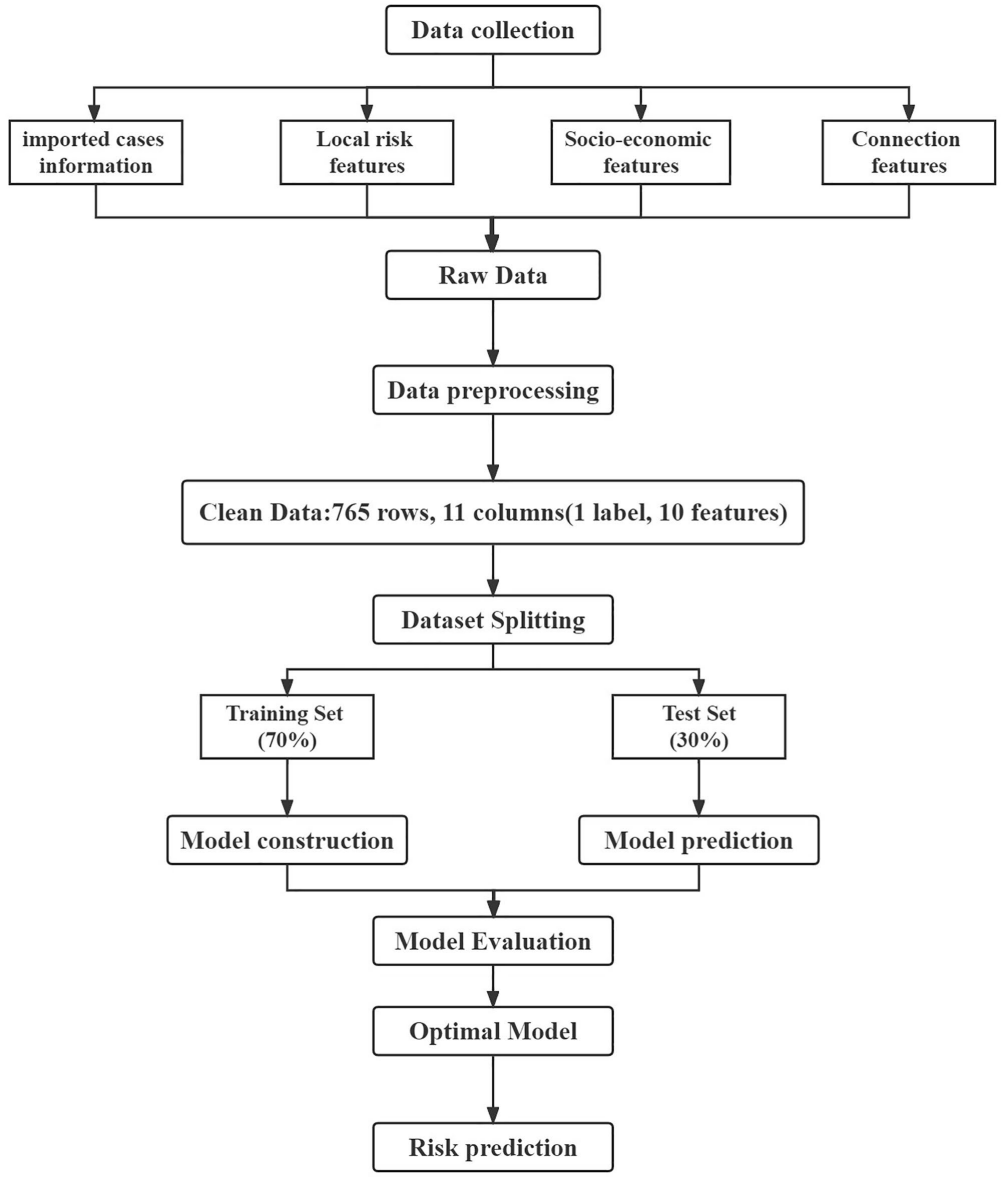
Research flowchart

## Data and method

The data preprocessing and modeling procedures were conducted using R (version 4.3.1).

### Study area

This study focuses on countries or regions that have imported malaria cases to China, and have established population and economic trade connections with China. The majority of these countries are situated in the regions of Africa and Southeast Asia. Annually, China records a total of more than two thousand cases of imported malaria originating from the aforementioned countries [24]. Hence, it is imperative to evaluate and anticipate the levels of risk associated with these nations in order to offer assistance for decision-making in the prevention and management of imported cases of malaria.

### Data source

The data on imported cases of malaria is sourced from the National Institute of Parasitic Diseases (NIPD) within the Chinese Center for Disease Control and Prevention (China CDC). The dataset comprises fundamental details, such as the country of origin and the year of occurrence for the imported cases. The indigenous malaria cases and malaria-related deaths in the countries of origin are sourced from the annual World Malaria Report, which is published by the World Health Organization (WHO)(https://www.who.int/teams/global-malaria-programme/reports). Population data for the origin countries is sourced from the United Nations Population Division (https://www.worldometers.info/world-population/population-by-country/). The per capita Gross Domestic Product (GDP) and Gross National Income (GNI) of the countries of origin were sourced from the Food and Agriculture Organization of the United Nations (FAO), available at https://www.fao.org/faostat/en/#data/MK. The data regarding the aggregate value of imports and exports between China and its partner countries, China’s revenue generated from contracted projects in those countries, and the quantity of Chinese workers dispatched to engage in labor cooperation in the respective source countries are obtained from the China Statistical Yearbook (https://data.stats.gov.cn/publish.htm?sort=1). The measurement of the geographical distance between the capitals of the two countries was derived from the Google Maps platform (https://www.google.com/maps).

### Data preprocessing

A dataset of 27,088 records of imported malaria cases in China from 2011 to 2019 was obtained from the Chinese Center for Disease Control and Prevention (CDC). The dataset was sorted by year and country of origin, resulting in 765 rows. We collected Indigenous-Cases, DMCs, and IRs for each record from the World Malaria Report, population data from the United Nations Population Division, and GDP and GNI data from FAO. Additionally, we obtained TCT, TET, and LD data for each record from the China Statistical Yearbook, and distance data from Google Maps. And finally, based on the number of import cases, a risk level classification is established for the origin countries. The classification criteria are divided into quartiles: Low (imported cases ≤ 3), Medium (imported cases between 3 and 50), and High (imported cases > 50) based on the number of imported cases. With this classification criteria, the 765 records can be classified into three classes: High Risk (118 records), Medium Risk (232 records), and Low Risk (415 records). After obtaining the risk level labels, irrelevant variables such as country names and years are removed for modeling purposes. All the variables used in model construction was shown in the Table 1.

The initial dataset exhibits missing values for variables including Indigenous-Cases, DMCs, TET, and LD. However, the percentage of missing values for these variables is less than 10%, thus there is no necessity to eliminate variables. The ‘missForest’ package in the R programming language (https://cran.r-project.org/package=missForest) is used to impute the missing values. The proposed methodology utilizes a random forest model and a variable swapping technique in an iterative manner to predict missing values until reaching convergence, thereby effectively imputing the missing values. One notable benefit of this approach lies in its capacity to effectively manage intricate relationships among multiple variables, all while avoiding the need for excessive assumptions or data manipulations [25]. We also found that there was a slight imbalance in our dataset, so we tried to deal with this phenomenon by performing the SMOTE method on the dataset, which increases the number of samples in a few categories by synthesising some samples in a few categories to improve the learning effect of the model for a few categories. The SMOTE method generates new synthetic samples by selecting samples from a few categories in the feature space and interpolating the values between them. This is achieved by using the ‘DMwR’ package in Rstudio to implement SMOTE.

**Table 1:** Variables used in model construction

Feature Name	Definition	Source
Risk	Import Risk rating	Segmented based on the Quartile of the number of imported malaria cases
Indigenous-Cases	Indigenous Malaria Cases of origin country	World Malaria Report
DMCs	Death Malaria Cases of origin country	
IR	Malaria Incidence Rate of origin country	
Population	The population of the origin country	United Nations Population Division
GDP	GDP Per Capita of origin country	FAO
GNI	GNI Per Capita of origin country	
TCT	Total Custom Trade with China	China Statistical Yearbook
TET	Total Engineering Trade with China	
LD	Labor Dispatch from China	
Distance	Distance between the two capitals	Google Map

### Dataset splitting

The dataset is partitioned using the ‘caret’ package in R (version 4.3) (https://cran.r-project.org/package=caret). The dataset has been divided into a training set, which comprises 70% of the data, and a testing set, which accounts for the remaining 30% of the data. The ‘caret’ package employs a random sampling technique to maintain the consistency of proportions between the training and testing sets, aligning them with the original dataset. This practice aids in mitigating the introduction of class disparities during the data partitioning procedure and diminishes bias in model assessment, thereby facilitating more precise performance estimation.

### Model construction

We have selected six machine learning algorithms widely used in fields such as infectious disease prediction: Random forest, AdaBoost, C5.0, KNN, SVM, XGBoost, which have been used to construct our malaria risk level prediction model.

Random forest algorithm is an ensemble learning algorithm that improves model performance by constructing multiple decision trees and integrating them together. It has the advantages of high accuracy, less overfitting, and is particularly suitable for complex classification and regression problems.

AdaBoost (Adaptive Boosting) is an ensemble learning algorithm that iteratively trains weak learners (usually decision trees) and assigns different weights to each learner, ultimately combining them into a strong learner. It has the characteristics of high accuracy and strong adaptability, but the disadvantage is that it is sensitive to noise.

As an improved version of the C4.5 algorithm, the C5.0 algorithm has the characteristic of high accuracy, can automatically handle missing values, and has an automatic pruning mechanism, which helps generate models with more generalization ability, but it requires more computing resources.

KNN (K-Nearest Neighbors) is a simple and easy to understand classification and regression algorithm that is suitable for multi classification problems. It performs well on small datasets and is suitable for imbalanced datasets, but its performance may decrease in high-dimensional spaces.

Support Vector Machine (SVM) is a powerful supervised learning algorithm that is particularly suitable for small samples, high-dimensional data, and nonlinear problems. It can solve multi classification problems through strategies such as One vs. One or One vs. All, and is effective for small sample data and suitable for high-dimensional spaces.

XGBoost (eXtreme Gradient Boosting) is a gradient boosting algorithm that is improved and optimized on the basis of gradient boosting trees. This algorithm can calculate the importance of features, help users understand which features in the model are most critical for prediction, and can automatically handle missing values without the need for additional processing by users. XGBoost performs well in many competitions and practical applications. Its efficient implementation and optimization make training on large-scale datasets relatively fast, but requires more computing resources.

Six machine learning models were trained based on the train dataset. The ‘randomForest’ package (https://cran.r-project.org/package=randomForest) was used to construct the Random Forest model. The ‘adabag’ package (https://cran.r-project.org/package=adabag) was used to build the AdaBoost model. The ‘C50’ package (https://cran.r-project.org/package=C50) was utilized to create the C5.0 model. The ‘class’ package (https://cran.r-project.org/package=class) was employed to develop the KNN model. The ‘e1071’ package (https://cran.r-project.org/package=e1071) was used to construct the SVM model. Lastly, the ‘xgboost’ package (https://cran.r-project.org/package=xgboost) was utilized to build the XGBoost model.

### Model evaluation

We utilized the six constructed machine learning models to make predictions on the test dataset. Confusion matrix was generated to visualize the prediction results. Model performance was evaluated by Accuracy (the proportion of correctly predicted samples out of the total samples), Balanced Accuracy (the average accuracy across all classes, taking into account class imbalances), Precision (the proportion of true positive predictions out of all positive predictions made by the model), Recall (the proportion of true positive predictions out of all actual positive samples), F1 score (the weighted harmonic mean of Precision and Recall, values closer to 1 indicating better model performance), and Kappa (a statistical measure of agreement between predicted and actual labels, Kappa values range from − 1 to 1, with values closer to 1 indicating better model performance). These evaluation metrics were then visualized and analyzed using the ‘ggplot2’ package (https://cran.r-project.org/package=ggplot2). Furthermore, for the two models that performed well, Random Forest (RF) and XGBoost, we computed and visualized their feature importance. The images for model evaluation were created using the ‘ggplot2’ package as well.

### Best model validation test

The best-performing model was employed to forecast the potential risk of malaria importation to China from various countries across the globe in the year 2019. A comparative analysis was performed using ArcGIS (version 10.8) software to visually compare the actual risk ratings and predicted risk ratings for the year 2019. The predicted risk levels were assessed against the observed situation.

## Results

### Dataset description

The Chinese Center for Disease Control and Prevention provided us with fundamental data regarding cases of imported malaria in China spanning the years 2011 to 2019. This dataset encompasses a total of 27,088 individual records. Upon categorizing the dataset according to the country of origin of the imported cases and the corresponding year, we acquired a dataset consisting of 765 rows and 11 columns. The data was partitioned into two sets: a training set comprising 70% of the data (537 rows, 11 columns) and a test set comprising the remaining 30% (228 rows, 11 columns). In this study, the variable “Risk” was designated as the label variable, while the remaining 10 columns (“Indigenous-Cases”, “DMCs”, “IR”, “Population”, “GDP”, “GNI”, “TCT”, “TET”, “LD”, “Distance”) were utilized as feature variables for the purpose of training six supervised learning models, including RF, AdaBoost, C5.0, KNN, SVM, and XGBoost.

### Model prediction and model evaluation results

**Fig. 2:**
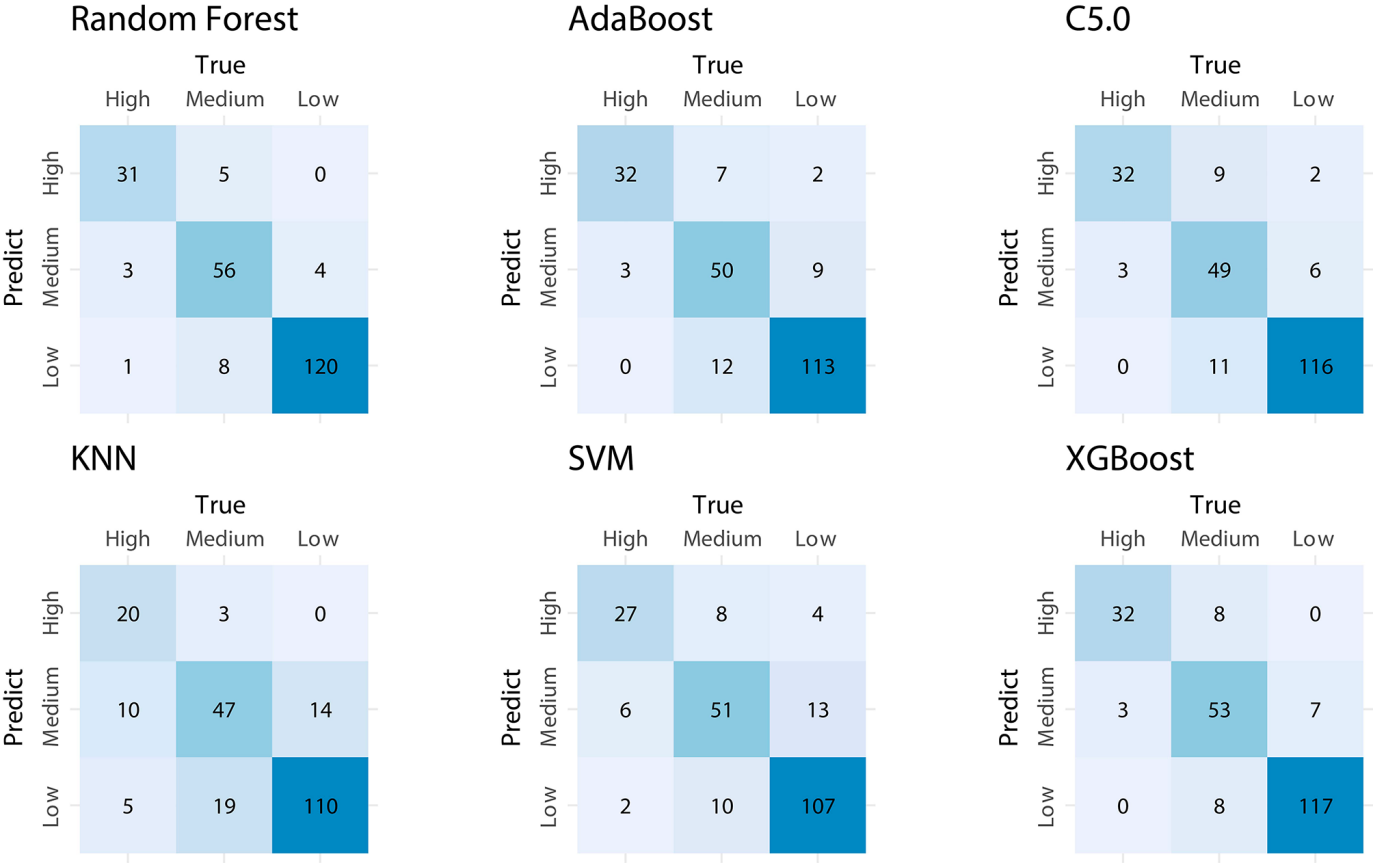
Confusion matrix of six models

**Fig. 3:**
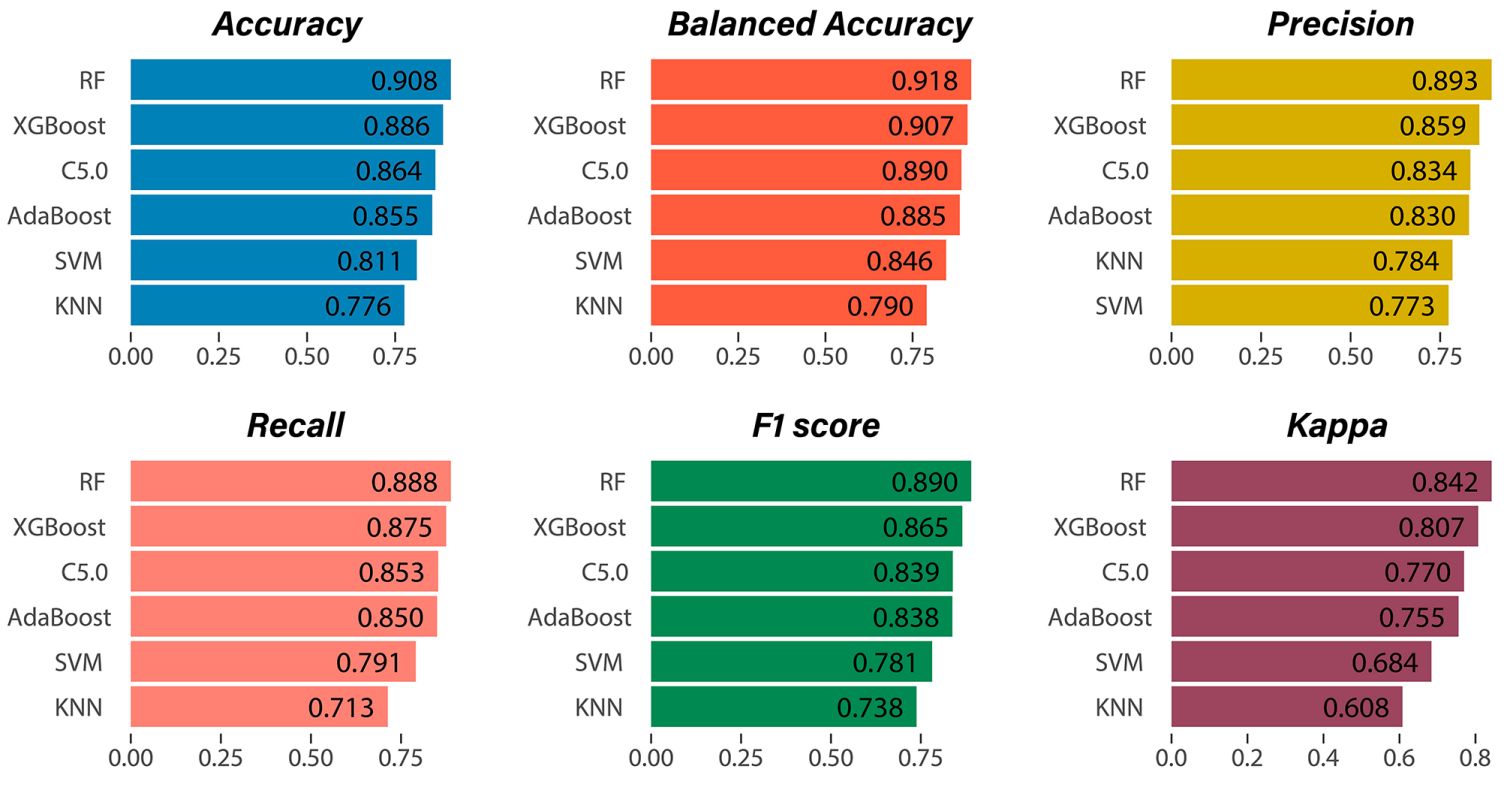
Model evaluation indicators

Utilizing the supervised learning models that were trained as previously mentioned, we conducted predictions on the test dataset. The visualization of the prediction results of the six models is depicted in separate confusion matrices, as illustrated in Fig. 1. Furthermore, an analysis of the evaluation metrics was conducted for the models, and the outcomes for the six models are depicted in Fig. 2. The Random Forest (RF) model demonstrates superior performance across all evaluation metrics, including Accuracy, Balanced-Accuracy, Precision, Recall, F1 score, and AUC. The Random Forest (RF) model demonstrated a notable level of accuracy, achieving a remarkable 90.8% on the test set. The XGBoost model exhibited exceptional performance, displaying evaluation metrics that were comparable to those of the RF model. The model evaluation results were shown in Fig. 3.

### Feature importance

**Fig. 4:**
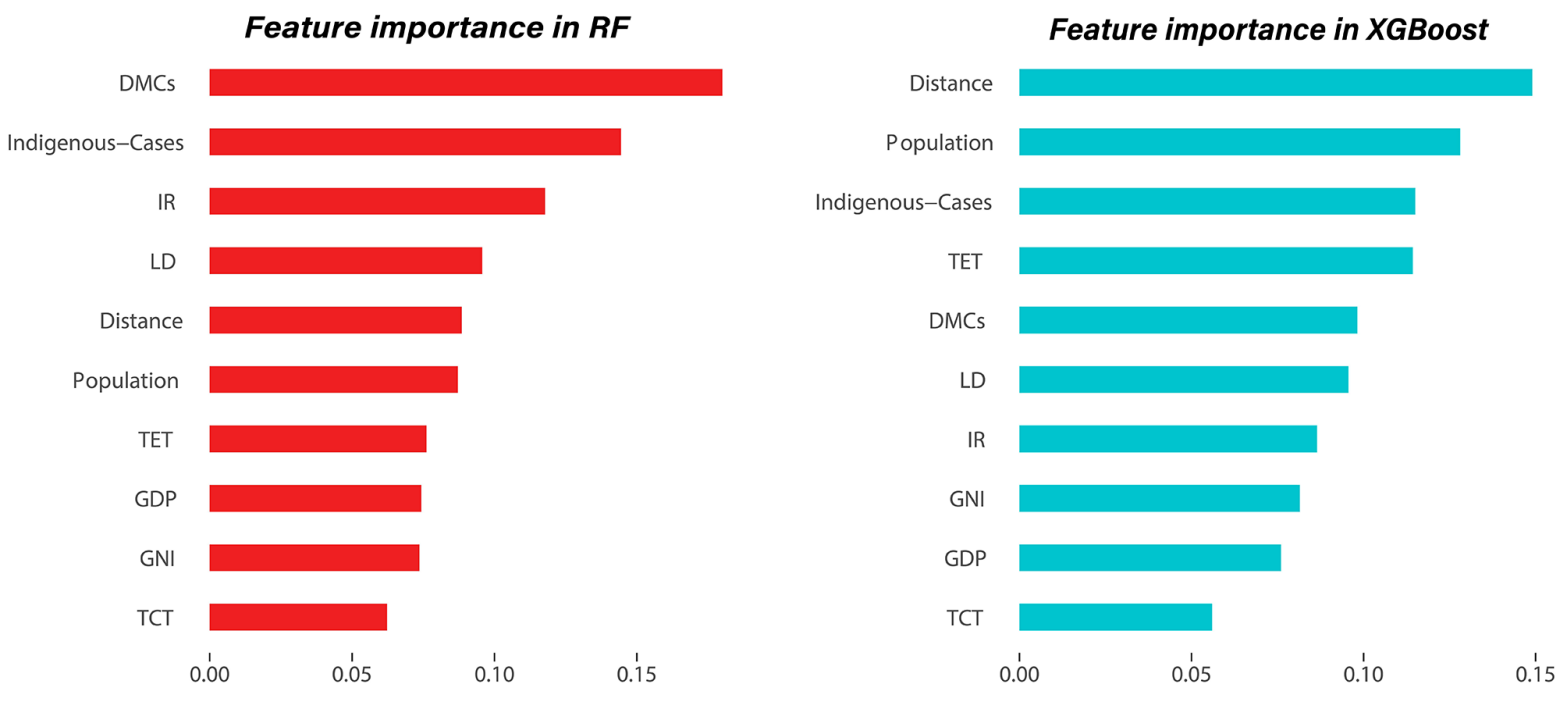
Feature importance of RF and XGBoost

Feature importance calculations and comparisons were carried out for the two models that exhibited superior performance in terms of evaluation metrics, namely Random Forest (RF) and XGBoost. The findings are illustrated in Fig. 4, which highlights variations in the rankings of feature importance between the two models. The Random Forest (RF) model assigns higher importance values to DMCs, Indigenous-Cases, and IR, while the XGBoost model assigns higher importance to Distance, Population, and Indigenous-Cases.

### RF prediction result

**Fig. 5:**
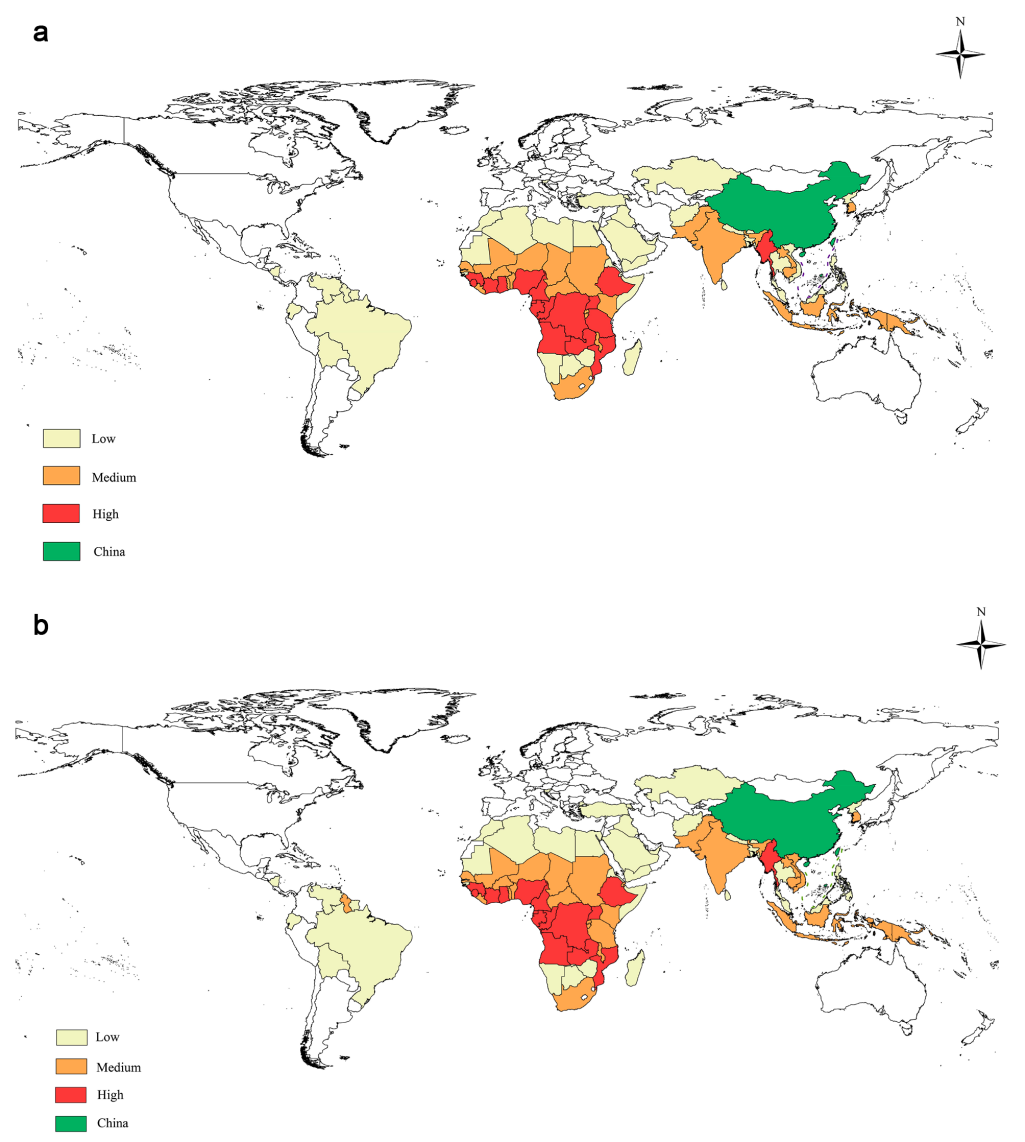
**(a)** 2019 Risk level of China imported malaria source countries (predicted by RF). **(b)** 2019 Risk level of China imported malaria source countries (actual situation)

We utilized the best-performing RF model from the six models to predict the risk level of the origin countries for imported malaria in China in 2019. We then compared the predictions with the actual risk from 2019. Using ArcGIS software (version 10.8), we visualized the predicted and actual results, as shown in Fig. 5(a) and Fig. 5(b) respectively. The RF model demonstrated excellent predictive performance, with only four misclassified regions. The accuracy achieved was 95.3%, indicating a strong model performance.

## Discussion

China’s notable accomplishments in the prevention and management of endemic malaria have attracted global recognition. Nevertheless, subsequent to the triumphant eradication of indigenous malaria, China is presently confronted with the predicament of imported malaria. Based on available reports, China documented a cumulative count of 10,085 instances of imported malaria between the years 2017 and 2021. The preponderance of these cases were traced back to Africa, accounting for 86.7% of the total, while Asia contributed to 12.4% [26, 27]. The risk factors pertaining to imported malaria may exhibit variations in comparison to those implicated in the transmission of malaria within local populations. Previous studies have predominantly concentrated on natural environmental factors, including rainfall, temperature, and vector capacity [28]. However, it is possible that these risk factors may have undergone alterations during the post-elimination phase. Hence, it is imperative to undertake a more comprehensive examination of factors such as population mobility and socio-economic dimensions.

This study approached the problem of predicting the risk levels in the source location of imported malaria in China from the perspectives of socio-economic factors and population mobility. By leveraging a dataset spanning nine years of historical records and employing advanced machine learning algorithms, a set of six distinct machine learning models were developed with the primary objective of accurately predicting the risk levels associated with the source location of imported malaria cases in China. These models offer novel perspectives and approaches for the evaluation of imported malaria risk in China. The Random Forest (RF) and XGBoost models exhibited superior performance based on the evaluation of six model metrics, including Accuracy, Balanced Accuracy, Precision, Recall, F1 score, and Kappa value. Both of these models demonstrated exceptional performance across all evaluation metrics. The RF and XGBoost models are categorized as ensemble learning algorithms, which involve the aggregation of multiple weak learners, such as decision trees or other base models. This approach enables the creation of a robust ensemble model that can effectively capture intricate relationships among features. In the process of constructing weak learners, both Random Forest and XGBoost utilize feature importance to facilitate feature selection, assess the significance of features, and allocate greater weights to influential features. This approach ultimately enhances the performance and generalization capability of the model. Additionally, it is worth noting that they also exhibit a certain level of resistance to overfitting. Hence, in terms of predicting the risk of imported malaria, the Random Forest and XGBoost models demonstrated superior performance when compared to the other four models [29–33].

In this study, the Random Forest model we constructed utilizes Gini importance to calculate the feature importance. Gini importance measures the importance of a feature by calculating the number of times that feature is used as a splitting node across all decision trees. In Random Forest, each decision tree selects the best splitting point based on the feature’s impurity [34]. The importance of a feature depends on its frequency of being used as a splitting node in multiple decision trees. The higher the frequency of a feature being used as a splitting node across more decision trees, the higher its Gini importance. Gini importance quantifies the contribution of a feature to the predicted results in the Random Forest model. When a feature has a higher Gini importance value, it indicates that the feature plays a more significant role in the model and has a greater impact on the predicted results [35]. In contrast, in the XGBoost model, feature importance is computed based on Cover, which represents the number of times a feature is used in the model. Cover measures the frequency of each feature being used in the model, specifically as a splitting node [36–37]. A higher Cover value indicates that the feature is more frequently used in the model, suggesting its greater importance.The difference in feature importance between the two models may be attributed to the nature of the algorithms they are based on and the distinct ways in which they learn. Random Forest and XGBoost are two distinct machine learning algorithms that vary in their data processing and pattern learning methods. Random Forest uses an integrated learning-based approach to make predictions by voting on multiple decision trees, while XGBoost is a gradient boosting algorithm that improves model performance by iteratively training weak learners. Random Forest and XGBoost are both machine learning algorithms used for making predictions. It is important to note that these two algorithms may have different preferences for feature selection and weight assignment. Feature importance indicates the most critical features for the prediction goal. In the case of the Random Forest model, it places greater emphasis on the prevalence of malaria in the country/area, as evidenced by the number of local malaria deaths, episodes, and incidence. This reflects the model’s focus on the severity of malaria in the source area. For the XGBoost model, the variables that contribute the most are the geographic distance of the two countries and the local population. This suggests that when controlling imported malaria, we should not only focus on the malaria prevalence in the area where the traveller originates from but also take into account local socio-economic factors such as the population.

We conducted empirical testing on the RF model, which demonstrated the best performance among the six models. The model was tested using historical data of the year 2019 to predict the imported malaria risk. The results showed an accuracy of 95.3% and a Kappa value of 0.923, with only four regions having incorrect risk predictions. These findings indicate that the RF model performs exceptionally well in prediction. To visually represent the prediction results, we created a risk map using ArcGIS software (version 10.8) and compared it with the actual results. Based on the predicted risk map, we observed that Central Africa and Southeast Asia are the regions with the highest risk of imported malaria for China, these results aligns with our existing knowledge of the situation, the previous research on the risk assessment of imported malaria in China showed that the main source countries of imported malaria in China are concentrated in Southeast Asia and Africa, with an average of about 2000 cases of imported malaria detected each year. The predicted risk level of imported malaria obtained in our study can be well matched with published research, proving that the risk level prediction model based on machine learning algorithms proposed in this study is reliable and effective [38–45]. It is important to acknowledge the limitations present in our study. Initially, it is crucial to recognize that the limitations of data availability may have led to the omission of certain pertinent socio-economic factors during the feature selection process. Consequently, this omission has the potential to compromise the dependability of our models. It is acknowledged that in subsequent research endeavors, the inclusion of additional pertinent variables will contribute to enhancing the precision and applicability of the models. Additionally, it is crucial to consider the ramifications of the COVID-19 pandemic spanning from 2019 to 2021, as it significantly influenced worldwide economic operations and patterns of human migration. Consequently, the machine learning models developed in this study are solely applicable for evaluating the risk factors associated with imported cases of malaria under the condition that population movements and economic exchanges are standardized. It is important to acknowledge that the effectiveness of the models may be constrained in situations of worldwide crises or other constraints [42].

## Conclusion

The findings of our study provide evidence for the efficacy of the Random Forest (RF) model as a valuable predictive tool in assessing the risk factors associated with imported cases of malaria in China. The model demonstrates strong performance across a range of evaluation metrics and demonstrates a significant level of concordance with empirical observations. The aforementioned findings hold substantial significance in informing the surveillance of imported malaria risk and offering methodological frameworks for evaluating the risk of other infectious diseases introduced from external sources. The utilization of the Random Forest model in the prediction of risk sources associated with imported malaria presents numerous advantages. The model successfully captures intricate interactions among features and enables the evaluation and selection of features. The identification of crucial features for assessing imported malaria risk can be achieved through the utilization of feature selection methods that are based on Gini importance. Additionally, the Random Forest model exhibits a notable degree of resilience against overfitting, thereby improving the model’s efficacy and ability to generalize on a broader scale. In conclusion, the findings of our research offer robust backing for the evaluation of imported malaria risk and serve as a valuable methodological resource for assessing the risk of other infectious diseases introduced from external sources. However, it is necessary to make further enhancements in our research, such as broadening the range of feature selection, taking into account additional socioeconomic factors, and conducting more thorough assessments of the model’s applicability.

## Data Availability

All data is public or available for application, and can be downloaded or requested from the corresponding author.

## Abbreviations

WHO: World Health Organization
China CDC: Chinese Center for Disease Control and Prevention
FAO: the Food and Agriculture Organization of the United Nations
RF: Random Forest
KNN: K-Nearest Neighbors
SVM: Support Vector Machine
ANN: artificial neural networks
GDP: Gross Domestic Product
GNI: Gross National Income
DMCs: Death Malaria Cases of origin country
IR: Malaria Incidence Rate of origin country
TCT: Total Custom Trade with China
TET: Total Engineering Trade with China
LD: Labor Dispatch from China
ArcGIS: Arc Geographic Information System
